# The Therapeutic Potential of *Phaseolus vulgaris* in Alzheimer’s Disease: Insights From Experimental Rodent Model

**DOI:** 10.1155/sci5/6739059

**Published:** 2026-07-14

**Authors:** Rabia Munawwar, Sana Sarfaraz, Rahila Ikram, Humera Anser

**Affiliations:** ^1^ Department of Pharmacology, Faculty of Pharmacy, Jinnah Sindh Medical University, Rafiqui H.J، Iqbal Shaheed Rd, Karachi, 75510, Pakistan, jsmu.edu.pk; ^2^ Department of Pharmacology, Faculty of Pharmacy, University of Karachi, Main University Rd 75270 NC-24 Deh Dih Korangi Creek, Karachi, 74900, Pakistan, uok.edu.pk

**Keywords:** Alzheimer’s disease, GC-MS, histopathology, liquid chromatography, red beans

## Abstract

Alzheimer’s disease (AD) is a devastating neurological ailment affecting millions of people worldwide. For this neurological disease, there are numerous pharmacological and supportive treatments available. Several organic products are currently being investigated for AD. In a recent study, a variety of methods were used to conduct research and evaluate this advantageous effect, including passive avoidance tests, histopathology, biogenic amine assessment, and GC‐MS analysis. For this study, three groups were made of albino rats of both genders. Group I was the negative control group (administered standard diet), Group II was treated with 500 mg/kg beans, and Group III was given 1000 mg/kg. All three groups were induced Alzheimer’s by giving 2.5 mg/kg diazepam prior to the start of the treatment. The results showed that a significantly higher dosage level of 1000 mg/kg was most effective at preventing dementia in AD‐induced rats as compared to the lower dose evaluated. Passive avoidance tests, GC‐MS analysis, which revealed acetylcholine activity and other beneficial compounds like squalene, isoglutamine, and pyrrolizine, as well as histopathological analysis, all supported the effectiveness of this nutritional option. The findings unmistakably demonstrated that 1000 mg/kg red beans are very helpful in reducing dementia in AD‐induced rats.

## 1. Introduction

Alzheimer’s is a neurodegenerative disease in which abnormal proteins deposit in the brain. The plaques and tangles in the brain are made up of amyloid and tau, which are typically soluble proteins that aggregate into amyloid‐like filaments. Tau inclusions can also be discovered in a variety of other diseases. Dementia can be due to the deposition of amyloid or tau malfunction [1]. The continuous molecular dissection of neurodegenerative pathways should lead to a complete understanding of Alzheimer’s disease causation [2]. There are many kinds of research that clearly validate the affirmative correlation between elevated serum cholesterol and the progression of Alzheimer’s disease in old age.

The prevalence of Alzheimer’s disease is more in women than in men. It is estimated that in 2050, the number of patients with dementia will increase from 5.8% to 13.8. The current study estimated that about 150 million people live with dementia around the world. Moreover, AD has become the fifth most significant cause of death worldwide [3].

In terms of the disease’s effects, Alzheimer’s disease is strongly linked to neurodegeneration and decreased cognition, including language abilities, praxis, loss of memory with loss of ability to recognize faces and recall names, loss of judgment and emotional stability, personality alterations, progressive and increased loss of neurons with the presence of senile plaques, neurofibrillary tangles, widespread neuronal network destruction, brain (cortex, hippocampus), and apparent hiatus [4].

According to some studies, the association of Alzheimer’s disease with aging suggests that the majority of older people have a high risk of developing it and that, given the prevalence of other dementias, almost everyone in their third age has the potential, but not necessarily, to develop some elderly disease, such as Parkinson’s disease [5]. There are two forms of AD prevalence: familiar and sporadic, both of which have the same clinical and nosological symptoms [6]. The sporadic kind is the most prevalent and occurs after 65 years, although the familiar type can present sooner. Intriguingly, in situations of chromosome 21 trisomy, Alzheimer’s disease can start around the age of 30 years [7]. In addition, current research recommends that norepinephrine in the locus coeruleus is linked to the protection of neurons during aging, and a decrease of norepinephrine can also lead to AD.

The new research is fundamental to natural supplements to cure neurological problems. Natural products are safe and easy to take. Beans are an essential component of a diet, containing minerals, vitamins, proteins, amino acids, and fiber. Red beans, scientifically also known as *Phaseolus vulgaris,* share one of the vital roles in the bean’s family. *Phaseolus vulgaris* is associated with the family Fabaceae [8]. Minerals (iron and zinc), proteins, and vitamins abound. Ironically, dietary fiber benefits the reduction of cholesterol absorption and the removal of lipids from the body, making it ideal for weight loss. It can lower blood sugar levels and be used as an antidiabetic drug. It plays a function in the formation of kidney stones and infections of the urinary tract. Other investigations have found that these beans are pretty beneficial in treating rheumatism. It also contains certain significant elements that can aid in developing and strengthening neurons [9]. However, there is not enough research on these topics.

The purpose of this study is to determine whether red beans have any impact on dementia and whether this knowledge will help people deal with neurological problems in the future. The utilization of natural remedies for the treatment of mental illnesses is required as it is currently having a global impact.

## 2. Methodology

The Alzheimer’s model was established using albino rats between 180 and 200 g. In the animal house of the Department of Pharmacology at the University of Karachi, rats were accommodated in their regular habitat of polypropylene rat cages. Rats were observed closely to confirm that food and water and all eco‐friendly factors such as temperature and humidity levels stayed consistent at 25°C ± 2°C and 50%–60%, correspondingly. A 12‐h day and night cycle was also accurately considered.

The strategies placed in Helsinki Resolution 1964 were monitored when handling animals. The study was authoritatively approved and permitted by the Institutional Board of Advanced Studies and Research by Resolution No. 31 (P).

A standard diet is given to the rodents which contain 70% carbohydrates, 30% proteins, and 10% fats. This is called standard chow died (NFD) diet [10].

### 2.1. Red Bean Identification and Purchasing

The red beans are obtained at the local market and stored at room temperature in a zip‐lock plastic bag. PVS‐01‐20 was assigned to the seeds by the Department of Pharmacognosy at the University of Karachi.

### 2.2. *Phaseolus vulgaris* Powder Preparation

The beans were thoroughly cleaned and rinsed with cold water after being picked out to remove any impurities. Once all the water had evaporated, the beans were boiled and dried. The dry beans were processed into powder using an electric grinder. The right dose pellets were made using a few drops of water.

### 2.3. Approval Based on Moral Principles

The full adherence to all animal laboratory care recommendations has been confirmed by all authors. Animals were kept in accordance with Canadian Council on Animal Care (CCAC) regulations, which included giving rat’s standard food and water in a clean environment with a 12‐h cycle for day and night.

### 2.4. Model Development and Dosing Schedule

The albino rats were divided into three groups. Each group consists of 6 rats. All three groups were administered a 2.5 mg/kg dose of diazepam (I.P.) prior to the treatment for inducing the Alzheimer’s model in rodents. Group I was considered as positive control group and was administered a standard diet. Group II was given a 500 mg/kg dose of *Phaseolus vulgaris*, while Group III was administered 1000 mg/kg of *Phaseolus vulgaris* orally once a day in the morning.

### 2.5. Passive Avoidance Apparatus

The device comprised of two compartments, the bigger one rectangular with a grid floor measuring 50 by 50 cm and 35 cm high wooden walls. It had a roof that was movable in both directions. A 6 by 6 cm aperture in one of the walls connects the main compartment to a shadowy smaller room. A 15 cm by 15 cm electrifiable grid, a continuous current stimulator, 15 cm‐high wooden walls, and an operable ceiling were all features of the smaller compartment. A sliding Plexiglass door was used to close the opening between the two sections. About 150 cm above the center, a 100‐W lamp provided illumination for the bigger compartment [11].

### 2.6. Acetylcholinesterase Levels Analysis

On the 12th day, a sample was taken in a Stat/Line pulls 3 mL green/yellow‐top (plasma separator) tube to measure the rodents’ acetylcholinesterase levels. After prepurification, the procedure used was immunoassay extraction; however, radioimmunoassay is the preferred method.

### 2.7. Hippocampus Evaluation Through Histology

On the 12th day, rodents were dissected, and the brains of the rats were taken out. The brains were then submitted to the lab for a gross inspection of the hippocampus, and slides were made to assess the neuronal alterations in the hippocampus of disease control and treated rats [12].

### 2.8. Liquid Chromatography for Biogenic Amines

Brains were homogenized with 5 mL perchloric acid for the extraction. The samples were continually shaken every 10 minutes while being refrigerated for one hour. The mixture was then filtered using Whatman filter paper (180 m thickness and 11 m particle retention rating at 98% efficiency) and then centrifuged at 1000 g for 10 min at 4°C. Then, the solution was injected into the HPLC, the mobile phase acetonitrile: water 42:58 [13].

### 2.9. Gas Chromatography Mass Spectroscopy

For GCMS, beans were crushed, dissolved in methanol‐based extracting solution (after being steeped in it for a few hours), and then placed in a sonicator for three hours to remove any remaining residues. The supernatant solution was then taken and added to a GCMS instrument for the evaluation of fatty acids and flavonoids. The process is also known as ultrasound extraction and analysis using selected ion monitoring (SIM) mode for GCMS.

### 2.10. Statistical Analysis

The statistical analysis used was SPSS Version 22. The two‐way ANOVA test and Turkey test were applied to measure the behavior evaluation.

## 3. Results

### 3.1. Passive Avoidance Apparatus Reading

Table 1 shows the effect of *Phaseolus vulgaris* in different doses (500 and 1000 mg/kg) on passive avoidance test in rats.

**TABLE 1:** Passive avoidance test readings.

Groups	0 day	7th day	9th day	12th day
Disease control	2.75 ± 5.44	23.0 ± 5.44	50 ± 5.44	31.0 ± 5.44
Red beans (500 mg)	7.0 ± 5.44	46.0 ± 5.44^∗∗^	30.2 ± 5.44^∗∗^	49.5 ± 5.44^∗∗^
Red beans (1000 mg)	7.5 ± 5.44	277.5 ± 5.44^∗,^, ^∗∗^	300 ± 5.44^∗,^, ^∗∗^	300 ± 5.44^∗,^, ^∗∗^

^∗^Comparison with 500 red beans with rest of the treated.

^∗∗^Comparison with disease control.

Post hoc analysis by Tukey’s test showed a highly significant (*p* ≤ 0.005) effect as compared to negative control throughout the treatment period by both doses.

Among the treated groups till Day 0, there was no significant difference in the results; however, on the 9th and 12th day, 1000 mg/kg showed a significant increase in time to enter the dark compartment as compared to disease control, but there is no significant difference noticed in 500 mg/kg dose.

### 3.2. Values of Acetylcholinesterase

**FIGURE 1:**
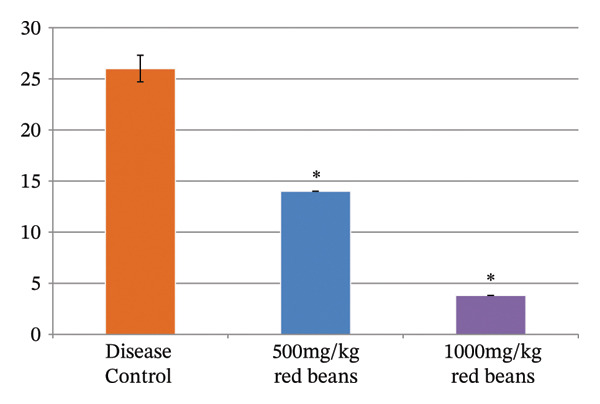
Acetylcholinesterase reading in blood of rats.

Figure 1 indicates the values of acetylcholinesterase in blood of rats. The value of acetylcholinesterase in disease control is much higher than the 1000 mg/kg red beans. It is also noticed that 500 mg/kg red beans have lower value than disease control.

### 3.3. Histopathological Evaluation of Hippocampus of Rats

Hippocampus of disease control shows edematous brain tissue with neurodegenerative changes as well as foci of vacuole degeneration.

500 mg/kg hippocampus shows edematous brain tissue with neurodegenerative changes. Foci of vacuolar degeneration are also appreciated, while in 1000 mg/kg, brain tissue shows foci of neuronal regeneration, mild edema, and scattered areas of neuron degeneration.

 Control: Brain tissue showing edema, neuro degeneration (thin arrow), and vacuolar degeneration (thick arrow)
 500 mg/kg: Photomicrograph 20X: Brain tissue showing edema, neuro degeneration (thin arrow), and vacuolar degeneration (thick arrow)
 1000 mg/kg: Photomicrograph 20X: Brain tissue shows foci of neuronal regeneration (thick arrow), mild edema, and scattered areas of neurodegeneration (thin arrow).

### 3.4. Histopathological Chart Indicating Changes in Control and Treated Groups

Table 2 shows the intensity of edema, vascular degeneration, neuro‐degeneration, and neuroregeneration in the hippocampus of rats.

**TABLE 2:** Histopathological chart.

Groups	Edema	Vascular degeneration	Neurodegeneration	Neuroregeneration
Disease control	+++	+++	+++	−
500 mg/kg red beans	+	++	++	−
1000 mg/kg red beans	+	+	+	++

*Note:* +++ indicates severe. ++ indicates moderate. + indicates mild. − indicates no change.

### 3.5. Liquid Chromatography for Biogenic Amines in Rat’s Brain

Table 3.

**TABLE 3:** Liquid chromatography for biogenic amines.

Peak no.	Retention time	Area (%)	Name
1	3.44	0.31	Performic acid
2	5.980	0.19	Acetylcholine bromide
3	18.4	0.41	Cyclotetrasiloxane
4	51.6	0.24	Furanone

## 4. Discussion

**FIGURE 2:**
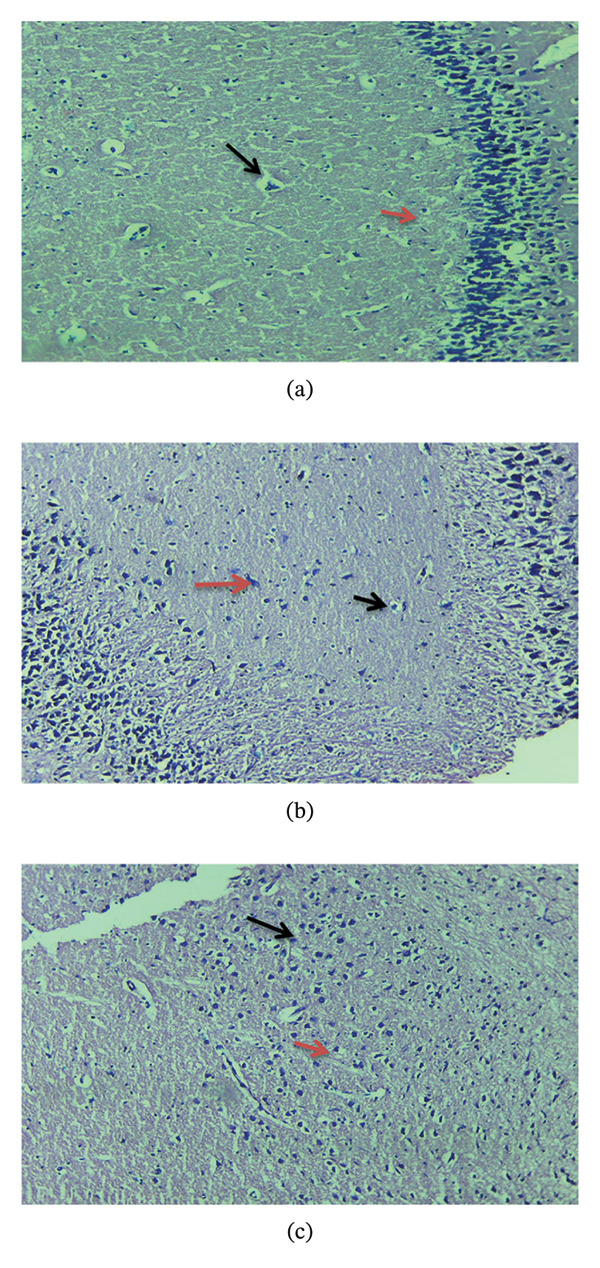
Histological slides of hippocampus of rat’s brain. (a) Disease control. (b) 500 mg/kg red beans. (c) 1000 mg/kg red beans.

It is well known that Alzheimer’s disease is due to the deposition of Aβ in brain parts which ultimately destroys neurons and causes learning impairment and cognitive disorders. In our study, it is observed in Figure 2 that the learning behavior of rats improved on the 9th and 12th day of the 1000 mg/kg dose. It is since squalene presents in red beans from a catalytic enzyme squalene epoxidase which is responsible for reducing cholesterol by producing neurochemicals which are analogs of neuronal chemicals, helps in transferring neurotransmitters and that is why squalene is known as anti‐Alzheimer’s agent [14].

The presence of isoglutamine in red beans also helps in improving memory through converting into glutamate by the enzyme isoglutamine synthase, and then it is converted into GABA with the help of vitamin B6 and with the enzyme glutamate decarboxylase. GABA helps in reducing the rate at which neurons fire and also help in preventing the overstimulation of neurons. So isoglutamine in red beans is indirectly helping restore memory and thus treating Alzheimer’s disease [15].

In the current study, it is noted in Figure 3 that in 1000 mg/kg, neurons are regenerating and also the edema in neuronal cells decreases; this is due to the pyrrolizine alkaloid present in red beans as mentioned in GC‐MS in memory loss. This pyrrolizine acts as a cholinesterase inhibitor which plays a key role in dementia as there is a loss of acetylcholine neurons in dementia [16]. Acetylcholine may improve memory retention by increasing the importance of feeding forward sensory nerves to the cortex and causing cortical circuits to respond to characteristics of sensory stimuli while lowering excitatory feedback activity and facilitating retrieval [17]. Thus, pyrrolizine acts as an anti‐Alzheimer’s agent by reducing acetylcholinesterase enzyme which ultimately increases the presence of acetylcholine in the synaptic cleft and helps in restoring memory.

FIGURE 3Presence of biogenic amines through in rat’s brain.

**Figure:**
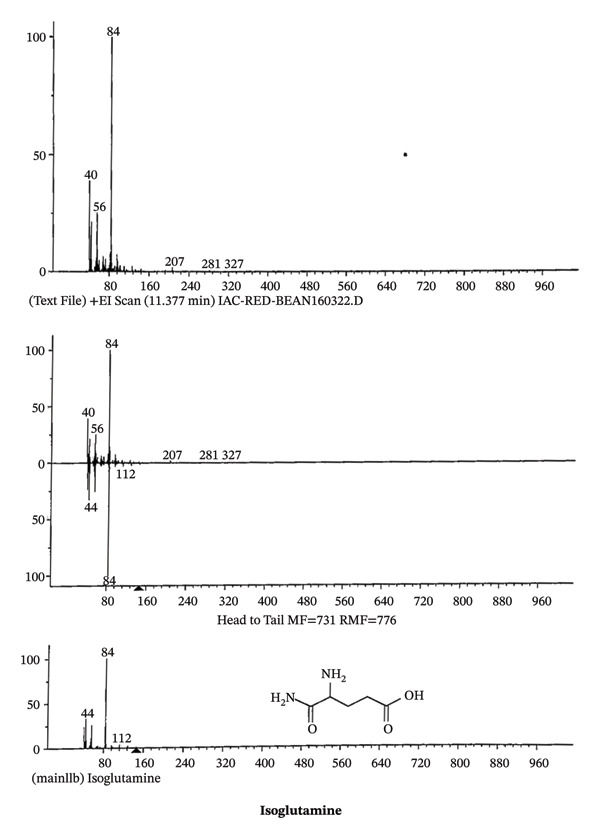

**Figure:**
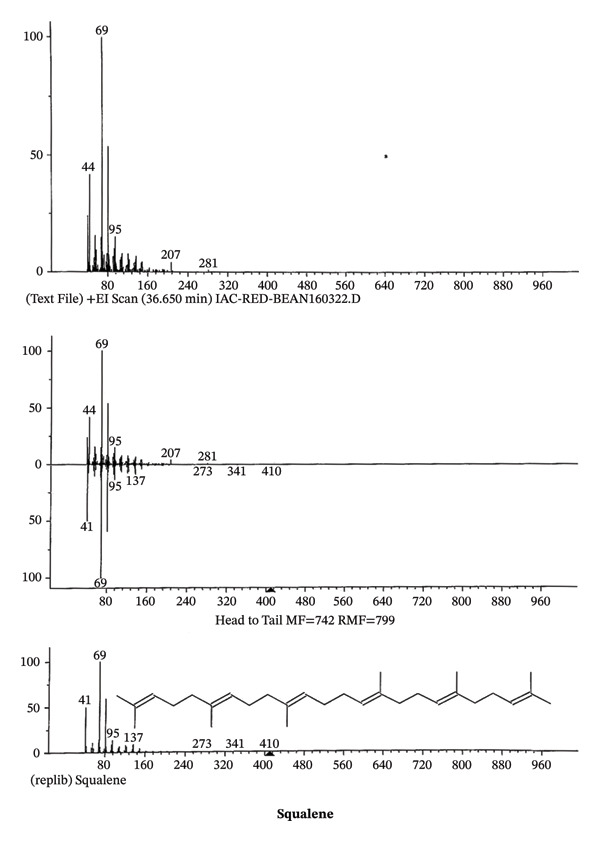

**Figure:**
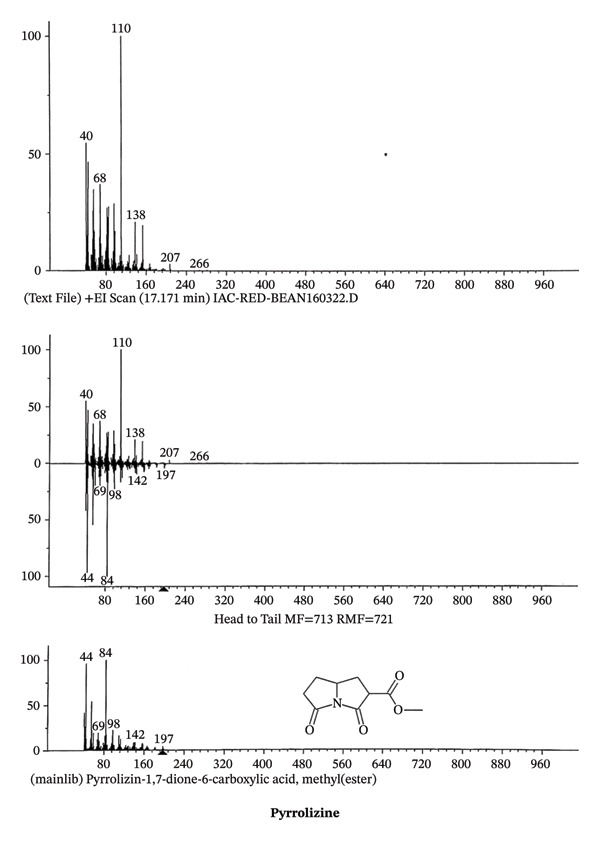

Alzheimer’s disease is highly affected by cacetylcholine; the more the acetylcholine, the more it helps in increasing connections between neurons and thus decreases the symptoms of dementia temporarily [18]. In this research, Figure 4 shows the brains of red bean rats showing the presence of acetylcholine bromide after liquid chromatography, which indicates that there is a high escalation of acetylcholine in treated rodents, thus decreasing the symptoms of dementia. Furanone also found in rat’s brains of red beans which is an antioxidative compound and helps in Alzheimer’s condition through neuron protective activity [19]. This compound is found more in 1000 mg/kg.

FIGURE 4Gas chromatography mass spectroscopy results.

**Figure:**
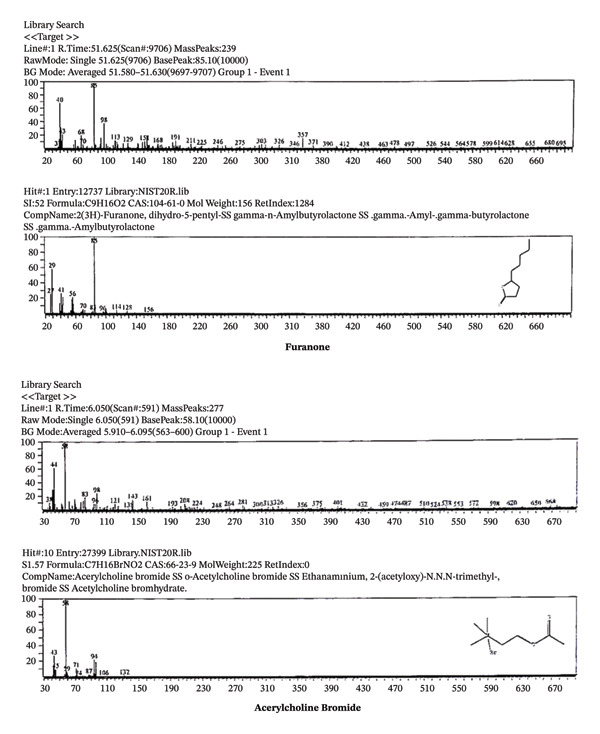

**Figure:**
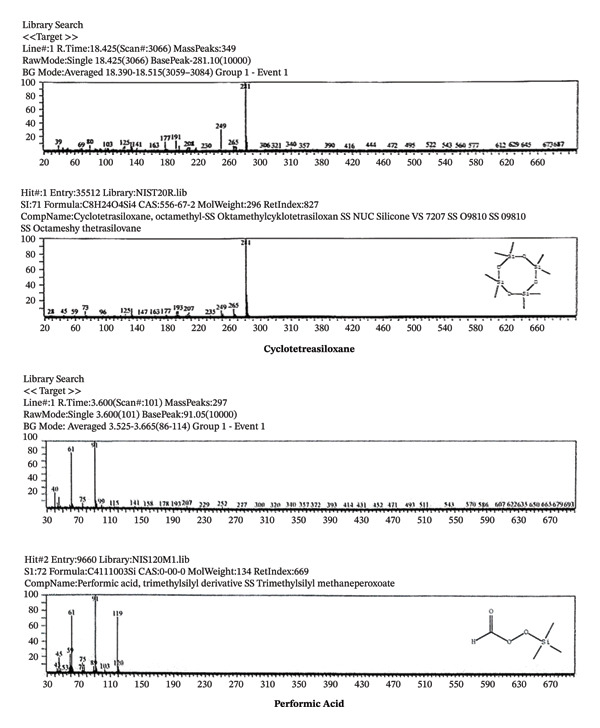

The decrease in acetylcholinesterase through blood testing showed a decrease in dementia by the mechanism that acetylcholinesterase is used to break the chain of the neurotransmitters acetate and choline; however, when its concentration is low, it will increase acetylcholine in the brain which increases learning ability and cognition [20].

Overall, the findings of this study indicate that red beans improved Alzheimer’s disease–related symptoms, as demonstrated by the passive avoidance test, acetylcholinesterase assay, histopathological evaluation of the hippocampus, and biogenic amine analysis using liquid chromatography. In addition, GC–MS analysis of red beans suggests that a dose of 1000 mg/kg may be beneficial in reducing symptoms associated with dementia.

## Funding

No funding was received for this manuscript.

## Ethics Statement

The research is approved by IRB on 29‐10‐2020 through a number resol (10) p 16.

## Conflicts of Interest

The authors declare no conflicts of interest.

## Data Availability

Data may be made available upon request.
